# Pathogenesis of HIV-associated depression: contributing factors and underlying mechanisms

**DOI:** 10.3389/fpsyt.2025.1557816

**Published:** 2025-04-17

**Authors:** Silvere D. Zaongo, Wenlin Wu, Yaokai Chen

**Affiliations:** ^1^ Department of Infectious Diseases, Chongqing Public Health Medical Center, Chongqing, China; ^2^ College of Public Health, Chongqing Medical University, Chongqing, China

**Keywords:** HIV, depression, factors, pathogenesis, mechanisms

## Abstract

Cumulative evidence indicates that compared to HIV negative individuals, people living with HIV (PLWH) have a higher likelihood of developing depression, anxiety, and cognitive disorders. Depression, which is known to be a persistent and overwhelming feeling of sadness accompanied by a loss of interest in usual activities, is one of the most common mental illnesses encountered during HIV infection. Experts believe that several factors such as neuroinflammation, life stressors, lack of sleep, poor nutritional state, opportunistic infections and comorbidities, and HIV medications are contributing factors favoring the development of depression in PLWH. However, the fundamental mechanisms which underlie the involvement of these factors in the emergence of depression in the context of HIV remain poorly explored. Past researches describing the role of one or two of the preceding factors do exist; however, very few articles tackle this important topic while considering the several different putative causative factors comprehensively in the particular context of HIV infection. Herein, we elaborate on the factors currently understood to be responsible for the development of depression, and discuss the particular fundamental mechanisms whereby each factor may result in the outcome of depression. We believe that the understanding of these factors and of their underlying mechanisms is essential for the development of future therapeutic interventions to alleviate the burden of depression commonly seen in PLWH, and therefore facilitate the development of strategies to improve their overall quality of life.

## 1 Introduction

Depression, also referred to as major depressive disorder or clinical depression, is a mood disorder that causes a persistent feeling of sadness and a loss of interest in usual activities. It is characterized by low mood, diminished self-worth, pessimistic thoughts, poor concentration, somatic symptoms (such as poor appetite, somnipathy, and fatigue), and increased withdrawal from social activities (1). Depression has the potential to lead to personal suffering, decreased productivity, increased health care costs, and a high risk of suicide. According to the World Health Organization (WHO), depression is the most prevalent psychiatric disorder in the world, with a global prevalence of 4% in 2017 (2). It is estimated that at least one in six people suffers from depression at some point in their lives (3), and according to the WHO, cases of anxiety and depression worldwide have increased by more than 25% as a direct consequence of the COVID-19 pandemic (4).

Depression is the most common comorbidity and neuropsychiatric complication in people living with HIV (PLWH) (5). This is due to the long-term effects of HIV infection itself (particularly the underlying chronic inflammatory state) (6) and the adverse effects of modern antiretroviral therapy (ART) on the central nervous system (CNS) (7), notwithstanding the fact that the widespread use of modern ART has significantly improved the survival and quality of life of PLWH (8). Past studies (9, 10) have shown that the prevalence of depression among PLWH exceeds that in the general population by a factor of three, thus further decreasing the already deteriorated overall quality of life of PLWH, and further augmenting the pre-existing psychological toll of the almost ubiquitous social rejection and stigmatization that this category of patient has had to endure. In one systematic review, Niu et al., analyzed 94 studies concerning the mental health of PLWH in China between 1998 and 2014, and observed that depression was highly prevalent in Chinese PLWH, with prevalence exceeding 60% in most included studies (11). In a more recent publication, the same team emphasized the need for improved screening of newly diagnosed HIV-positive individuals, as at least 10% of these individuals have persistent depression (12). It has been reported that the tandem of HIV-associated co-morbidity with depression often leads to longer-lasting depression and more severe depressive symptoms, such as a greater degree of mental stress and low self-esteem, decreased appetite, poor sleep quality, and a higher prevalence of Major Depressive Disorder (MDD) (13). In another systematic review, Lofgren et al., pooled 70 research articles from 16 African countries for analysis, and observed that the prevalence of MDD in Sub-Saharan PLWH was 15.3% (14). Notably, it was believed that the prevalence of depression in PLWH differs between high-income and low-income countries. Indeed, wealthy countries like Canada and the US have prevalence rates of 20-40% (15, 16). Differences in the observed prevalence of depression between wealthy countries and low-income countries (such as those in Sub-Saharan Africa) may be a consequence of their under-representation in the literature, poor access to care, lower awareness of depression, and the stigma associated with depression, amongst other factors (17–19). Otherwise, prevalence rates of between 20-40% have been reported in countries such as Cameroon (26.7%) (20) and Ethiopia (36.65%) (21).

It is generally accepted that the etiology of depression is associated with many factors, and includes genetic factors, neuroendocrine factors, neuroimmune factors, and changes in concentrations of neurotransmitters (22). Up until now, several hypotheses, such as the hypothalamus-pituitary-adrenal (HPA) hypothesis (23), the cytokine hypothesis (24), and the monoamine neurotransmitter hypothesis (25) have all been proposed as the basis for the pathogenesis of depression. However, it is worth noting that the pathogenic mechanisms underlying the emergence of depression in the context of HIV infection remains sparsely researched, as this investigational realm is, and has been, challenging as a consequence of the diverse cultural considerations that need to be addressed in the assessment of depression, and also the diverse variety of investigational methods and tools used for its diagnosis (**Table 1**). These factors may certainly represent etiological clues; however, it is generally accepted that additional factors and the specific anatomical states induced by HIV infection, such as the persistent underlying chronic inflammation (particularly in the brain, and consequences on neuroendocrine factors, neuroimmune factors, cytokine and neurotransmitter expression, etc.), HIV medication, the lack of sleep, poor nutrition, etc., may be considered as further etiological elements as well. In this article, we provide an extensive review of factors favoring the emergence of depression (as reported in contemporary literature), and the manner in which the association of these factors with HIV infection materially increases the likelihood for the onset of depression in PLWH.

**Table 1:** Contemporary depression assessment tools.

Category	Tool	Description	Ref
Across the lifespan	Beck depression inventory	Is widely used to screen for depression. It is also used to measure behavioral manifestations and severity of depression	(26–28)
	Center for epidemiologic studies depression scale	While originally designed for use in the general population, this tool is now used to screen for depression in primary care settings.	(29, 30)
	EQ-5D	Introduced by European researchers (EuroQol group), it measures the quality of life in five dimensions (5D), viz., mobility, self-care, usual activities, pain/discomfort, and anxiety/depression.	(31–33)
	Hamilton depression rating scale	Measures depression in individuals before, during, and after treatment.	(34, 35)
	Montgomery-Asberg depression rating scale	Measures the severity of depression in 18-year-old individuals or older.	(36, 37)
	Social problem-solving inventory-revised	Is a self-report measure of social problem-solving strengths and weaknesses in 13-year-old individuals or older	(38, 39)
Children and adolescents	Behavior assessment system for children	Assesses emotional disorders, personality constructs, and behavioral problems in people from 2-21 years old.	(40, 41)
	Child behavior checklist	Is designed and administered to parents to assess their children and adolescents’ (between 6 and 18 years old) social competence and behavioral problems.	(42, 43)
	Children’s depression inventory	Is an adaptation of the Beck depression inventory for adults, and used in children. It assesses depression severity in children and adolescents aged 7 to 17 years old.	(44, 45)
	Children’s depression rating scale	Originally designed to measure depressive symptoms in children (6-12 years old), it is also used in the adolescent population	(46, 47)
General adult population	Beck hopelessness scale	Assesses an individual’s (17 to 80 years old) negative expectations about the future.	(48)
	Quick inventory of depressive symptomatology-self-report	Measures the severity of depressive symptoms in adults (≥18 years old).	(49)
	Patient health questionnaire	Is used to screen depressive symptoms	(50, 51)
	Reminiscence functions scale	Assesses the frequency with which adults (≥18 years old) engage in the act of recollecting past experiences (or events).	(52, 53)
	Short form health survey	Is a self-report of patients which measures quality of life and care outcomes.	(54)
	Social adjustment scale-self report	Is a self-report measuring social functioning in adults.	(55, 56)
	Social functioning questionnaire	Is a self-report measuring social functioning in adults over the last two weeks.	(57)
Older adults	Geriatric depression scale	Screens and measures depression in adults	(58, 59)
	Life satisfaction index	Measures well-being and successful aging in adults older than 50 years of age.	(60, 61)

Ref, references.

## 2 Genetic factors

### 2.1 Evidence

Although depression is not a hereditary disease, it does possess a genetic tendency. In 2000, one meta-analysis analyzed five twin family studies and observed that depression has a family aggregation, and a heritability of 37% (62). The quality of the analyzed studies was robust; however, the potential impact of the environment on individuals included in each study was not considered. In 2006, a twin study with a very large sample size was conducted in Sweden (63). Through personal interviews with 42,161 individual twins (including 15,493 complete pairs), the prevalence of depression was assessed by utilizing the Diagnostic and Statistical Manual of Mental Disorders-IV (DSM-IV) diagnostic criteria. Their results indicated that the heritability of depression was 38%. Subsequently in 2014, a family study (64), which included 447 probands and 2082 of their relatives, reached a similar conclusion, i.e., that major depression exhibits a family aggregation (OR=2.26; 1.58-3.22; h2 = 0.20). Tansey et al. (65), estimated the contribution of common genetic variations to antidepressant response using Genome-Wide Complex Trait Analysis in a combined sample of 2799 antidepressant-treated subjects with major depressive disorder and genome-wide genotypic data. Their observation was that common genetic variants explain 42% of individual differences in antidepressant response (SE=0.180, *p*=0.009). The observations of the preceding studies thus confirm the familial aggregation of depression; however, from the perspective of HIV infection, this question remains to be further clarified. In order to obtain a clearer picture with respect to the specific case of PLWH with depression, we believe that a concise review of the contemporary literature related to genome-wide association studies (GWAS) and genome wide transcriptomic analysis is necessary.

### 2.2 Genome-wide transcriptomic analysis and GWAS in HIV-associated depression

It is known that HIV infection can be transmitted vertically from mother to child (66); however, this does not mean that HIV is heritable, nor that HIV is a genetic disease, as vertical transmission does not involve alterations to genetic material. Therefore, it is not logical or appropriate to search for relationships between HIV and depression on the basis of classical genetics. The emergence of the field of epigenetics thus provides us with a novel and exciting avenue from which to approach this subject. The preceding field of study, by definition, describes heritable changes in gene expression that are independent of changes in the regulated DNA sequence itself. Although DNA methylation and histone modification are two mechanisms that mediate epigenetic modification, noncoding microRNAs (miRs) are thought to be associated with epigenetic modification in two ways (67). Thus, miRs may induce DNA methylation either by interacting with the messenger RNA (mRNA) [miR-165 and miR-166 interact with PHABULOSA (PHB) mRNA to alter the chromatin of the template PHB gene (68)] or by targeting key DNA methylation enzymes (examples: DNMT1, 3a, and 3b) (69). In one systematic analysis by Juzwik et al., it was proposed that noncoding RNA disorders may promote the development of neurocognitive-related diseases, and especially that miRs affect transcription by stabilizing mRNA and preventing translation (70). In the particular case of HIV infection, our group has reported that miRs play a major role in HIV immunopathogenesis (by increasing or decreasing HIV replication) (71), and thus a closer look at the role of miRs in the development of HIV-associated depression is warranted.

Genome-wide transcriptomic analytic techniques, such as microarrays and RNA-seq, can identify changes in miRs and other noncoding RNAs. However, only a few studies have used human brain samples to study changes in miRs during HIV infection. The earliest research related to this can be traced back to 2008, when Eletto et al., using mice with HIV encephalopathy, identified a mechanism whereby HIV tat disrupts neuronal activity i.e., blocks the regulation of the level of expression of miRs (including mir-128) in primary cortical neurons (72). During this period, Mukerjee et al., using the Western blot technique and immunohistochemical analysis, observed that HIV-1 viral protein R (Vpr) plays an important role in neuronal dysfunction by regulating the level (upregulation or downregulation) of several miRs (for example miR-34a was found to be upregulated) and their specific target genes (e.g., CREB, the target gene of miR-34a), which may lead to the development of neurocognitive impairment (73). Yelamanchili et al., observed that miR-21, miR-142-3p, and miR142-5p are significantly dysregulated in the caudate nucleus and hippocampus and striatum brain samples of PLWH, and in SIV-infected rhesus monkeys (74). Subsequently, Xu et al., observed 17 significantly altered miRNAs in the brain tissues of patients with HIV-1 associated neurocognitive disorder (HAND), and compared them with miRNA samples from HIV positive individuals without HAND (6). Their results indicated the levels of miR-500a-5p, miR-34c-3p, miR-93-3p, and miR-381-3p were not only elevated in the brain of patients with HAND, but are also elevated in PLWH who do not have HAND (6). The expression of these specific miRNAs in PLWH will lower the level of peroxisome protein, which may lead to neuronal dysfunction in the CNS as demonstrated by a previous research team (75).

In addition to the study of epigenetics, candidate gene study and GWAS also point out another direction from which to study HIV-associated neurological diseases. This focuses mainly on the research of SNPs and genes related to the mutation of genes associated with the immune system. One case-control study comprising 86 HIV-associated depression cases and 246 non-HIV-associated depression cases tested seven candidate gene polymorphisms related to HIV-associated depression. Results from the study indicate that the CCR5 wt/Δ32 genotype is related to the duration of disease; however, the SNP in the candidate gene PREP1 was significantly different among the genotypes of all study cases and the control group (76). Interestingly, recent research shows that CCR5-Δ32 mutations may lead to the failure of HIV-1 gp120 to effectively bind to CCR5, thus preventing HIV from entering host cells (77). There are also a number of candidate gene studies (78) which focus on the genetic variations of TNFα and dopaminergic genes. However, it seems that these only play a role in HIV-associated neurocognitive disorders and HIV-associated dementia. Moreover, the sample populations in these studies were not specifically diagnosed with HIV-associated depression as a primary diagnosis. However, it is known that dopamine and TNFα levels also play important roles in the occurrence and development of depression. It is regrettable that only a small number of GWAS in HIV-related fields focus on HAND, and thus far GWAS related to HIV-associated depression have not, as yet, been published.

## 3 Neuroendocrine factors

### 3.1 The HPA axis and the HPA hypothesis for depression

Other than genetic factors, neuroendocrine factors should also be considered in the etiology of depression. Indeed, the HPA axis, which consists of the hypothalamus, the pituitary, and the adrenal glands [which regulates the production of glucocorticoids (GC)], has been implicated in the pathophysiology of anxiety and depression, as well as in the everyday processes of normal cognitive functioning (79). The HPA hypothesis suggests that depression is caused by hyperactivity of the HPA axis. When depression occurs in an individual, the sensitivity of the glucocorticoid receptor (GR) is impaired, which leads to an inhibition of the negative feedback mechanism regulating corticotropin-releasing hormone (CRH) secretion, which consequently leads to excessive central secretion of CRH, and thus increased circulating GC (80). It is worth mentioning that the sensitivity of the GR is basically regulated by the FKBP5 gene, encoding the FKBP51 gene, a co-chaperone of heat-shock protein 90 (hsp90). When FKBP51 binds to the GR, the affinity of glucocorticoid binding decreases, and the efficiency of GR transport to the nucleus decreases. FKBP5 mRNA and protein expression are induced by GR activation, and provide a negative feedback loop for GR sensitivity (81). At a cellular level, these genetic variations, combined with epigenetic changes, may explain structural and functional changes in several brain regions (82). When the body is in a state of excessive activation of the HPA axis, the likelihood of suffering from depression will also be substantially increased, because under normal conditions, lower GC levels interact with cortical and limbic structures (such as the prefrontal cortex and the hippocampus) to promote cognitive function and emotional processing. However, extensive research has shown that during the imbalance of homeostasis seen in chronic stress, a series of neurobiochemical changes (such as excessive GC production and hyperactivation of GR) result in reduced neurogenesis and neuroplasticity, especially in the hippocampus (83, 84). Thus, radiographic studies have shown that morphological changes occur in the hippocampus of patients with depression, and that the extent of hippocampal change positively correlates with the clinical course of depression (85).

### 3.2 The HPA axis in PLWH

HIV infection causes dysfunction in multiple somatic systems, including in the HPA axis. AIDS/HIV reduces host immune activity and alters cellular biological pathways through HIV encoded proteins, directly and indirectly affecting the HPA axis via the ‘stress’ represented by, amongst others, the ever-present opportunistic infections associated with the chronic immunodeficient state present in these patients, and due to the various adverse effects of the therapeutic compounds used to treat patients with HIV infection and AIDS (86).

From a pathophysiological perspective, the hypothalamus, the pituitary gland, and the adrenal gland all develop functional damage in PLWH. Firstly, the adrenal glands are often adversely affected in PLWH, and these patients often experience symptoms such as weakness, fatigue, and weight loss, which are symptoms that are similar to those seen in patients with chronic adrenal insufficiency (87). It is known that during early HIV infection, adrenal stimulation with adrenocorticotropic hormone (ACTH) induces an inadequate response in 14% of PLWH. The proportion with an inadequate GC response is further reduced in AIDS patients (54%) (87–89), suggesting progressive damage of the adrenal glands which is initiated at the early stage of HIV infection, and which further deteriorates during the AIDS stage. This is further corroborated at autopsy, where the adrenal glands are often found to be damaged in HIV/AIDS patients, mainly due to infections by opportunistic pathogens (e.g., *Cytomegalovirus*, *Mycobacterium, Cryptococcus. Toxoplasmosis, and Pneumocystis*) rather than by HIV itself, and which is facilitated by the profound immune deficiency ubiquitously present in PLWH at the AIDS stage of HIV infection (90). These opportunistic infections may be caused by the pathogens described, but may also encompass malignant tumors (such as non-Hodgkin’s lymphoma and Kaposi’s sarcoma) (91). In one study conducted in Nigeria in which 113 PLWH were recruited, the prevalence of adrenal insufficiency among PLWH was observed to be as high as 34% (92). During recent times, the incident rate of adrenal inflammation has declined significantly, as the inherent immunological functioning of PLWH has been observed to be more effectively preserved and the incidence of severe opportunistic infections has been observed to have decreased significantly, due to the widespread use of modern ART (93). With respect to the pituitary gland, it seems that the functional secretion of ACTH is generally retained in HIV/AIDS patients. As such, in one large study of 350 PLWH, 30.9% of the participants had serum cortisol levels below 100μg/L; however, after the administration of 1μg of ACTH, only 16.3% of subjects were observed to have serum cortisol levels below 180 μg/L (94). Then, in another study, autopsies were performed on 88 AIDS patients who succumbed to the disease, and only ten patients were observed to have extensive necrosis and/or focal fibrosis in the anterior pituitary (95).

Relative to the hypothalamus and pituitary gland components of the HPA axis, the adrenal glands sustain the most severe damage secondary to HIV infection. In chronic adrenocortical hypofunction/insufficiency, endocrine abnormalities are particularly serious, and may have a significant impact on the mental state of patients in the later stages of the disease, resulting in depression (96). When patients are diagnosed with adrenal insufficiency, most patients will choose long-term glucocorticoid treatment (97). As early as 1992, Norbiato et al., conducted a study that included nine PLWH with Cushing’s syndrome (98). In these patients, the affinity of the GR and its ligand in peripheral leukocytes decreased significantly, and the number of receptors increased, indicating that adrenal cortical dysfunction in these patients may have been caused by decreased sensitivity of peripheral tissues to glucocorticoids. Similar conclusions were also presented in another study (99). However, there are few recent such reports, and this may indicate that this situation is, in itself, rare, or may be related to an unknown cryptic mechanism. Nevertheless, it has been reliably demonstrated that glucocorticoid levels are significantly elevated in HIV patients with Cushing’s syndrome (100).

From a psychological perspective, PLWH are known to inherently be prone to stressful social circumstances, such as social isolation, discrimination, stigmatization, and other negative psychological emotions (101). Additionally, among PLWH (who, broadly speaking, tend to be economically vulnerable, tend to belong to ethnic minorities, tend to belong to sexual minorities, tend to be recreational drug users, and tend to be sex workers), the proportion of people belonging to socially vulnerable groups is high, and these individuals have an inherently greater risk of developing depression even prior to becoming infected with HIV (102). These ‘negative’ circumstances may activate the CNS to generate a series of stress responses, and this process is mainly mediated through the HPA axis (103). The HPA axis thus acts as a medium for the body to react to sources of stress. The end point of this endocrine pathway is the release of cortisol from the adrenal cortex. As a consequence, in these patients, the HPA axis may remain in an activated state for an extended period of time, and during this extended period of time, excessive glucocorticoids are produced and circulate within the body.

In PLWH, it is both the compensatory increase in production of glucocorticoids caused by adrenal insufficiency and the long-term and enduring negative life circumstances and events that lead to the overactivation of the HPA axis, and it is this overactivation that ultimately leads to the increased levels of circulating glucocorticoids. As mentioned above, glucocorticoids circulating in the body at an elevated level for an extended period of time will have a much greater impact on the CNS of the human body (especially in the hippocampus) (104), and may also predispose individuals to be more likely to develop depression. Future investigations will be necessary to clarify the preceding hypothesis and its fundamental mechanisms.

## 4 Neuroimmune factors

### 4.1 The cytokine hypothesis

Over recent years, researchers have observed that depression is associated with other chronic inflammatory and autoimmune diseases (105). As such, the cytokine hypothesis is an important theory that offers a further explanation for the pathogenesis of depression. The occurrence of depression is often accompanied by inflammatory responses, and the body’s baseline immunological functioning is thus altered by the presence of depression (106). Most studies concerning the mechanisms whereby an increase in cytokine levels causes depression focus on three likely explanations. Firstly, the release of proinflammatory cytokines may stimulate the HPA axis to secrete GCs (107). The proinflammatory effect of GCs is considered to be the basis of neurodegeneration (108). This is singularly important in the hippocampus, which is a brain region that controls cognitive function, and is an area of the brain which is particularly vulnerable to neuroinflammation and neurodegeneration (109). Secondly, on the one hand, cytokines may alter serotonin transporter (SERT) levels, and the consequent lower concentrations of serotonin induce the body to be more prone to anxiety and major depressive disorder (110). On the other hand, cytokines may activate indoleamine 2,3 dioxygenase (IDO) and increase 5-HT consumption through the kynurenine pathway, thus reducing 5-HT levels by negatively influencing tryptophan metabolism (111, 112). Thirdly, cytokines may materially affect neural plasticity by reducing the production of neurons, promoting the death of cortical nerves, and reducing the expression of brain-derived neurotrophic factor (BDNF) in the limbic system of the brain (113). Carlos et al., have utilized an *in vitro* approach in primary hippocampal neurons, and have observed that in the prolonged presence of IL-1β, BDNF is unable to efficiently deliver long-distance signaling via the retrograde transport of signaling endosomes (114). Thus, neural plasticity will be adversely affected.

### 4.2 Cytokines in HIV-associated depression

During the first few weeks of HIV infection, HIV may enter the CNS via a “Trojan horse” mechanism that permits infected immune cells to penetrate the blood-brain barrier (115). In conjunction with the trojan horse method, it is recognized that transcytosis, which is defined by the transport of HIV particles from the blood to the brain via epithelial cells, may result in CNS invasion by HIV (116). This allows HIV-activated monocytes, microglia, and other infected immune system cells to release viral proteins and infectious particles within the CNS, and subsequently promotes the inflammatory reaction, which substantially encourages the release of cytokines, and leads to the manifestation of a “sickness behavior” characterized by insomnia, loss of appetite, anhedonia, and difficulties with memory and concentration (117, 118). In order to counteract this, the body will activate the sympathetic adrenal medullary axis and the HPA axis (119). Thus, the activation of the HPA axis may be seen as an adaptive response to inflammation. For acute diseases, the “sickness behavior” subsides when the inflammation decreases and eventually dissipates. However, for chronic diseases such as HIV infection, the burden of HIV viral infection does not reduce. This leads to persistent clinical symptoms of chronic disease, and constitutes a significant obstacle to patient rehabilitation. Saloner et al. (120), recruited 143 PLWH to explore the relationship between depressive symptoms and inflammation, and observed that PLWH with depressive symptoms may be afflicted with an inflammatory subtype of depression, and may be particularly susceptible to neurocognitive changes.

#### 4.2.1 Interleukin-1 beta

Elevated levels of IL-1B are associated with depressive symptoms. In a study by Uint et al., researchers analyzed the plasma cytokine concentrations of 34 patients with depression, 43 patients with bipolar affective disorder, and 41 controls (121). It was observed that, compared to those with bipolar disorder and the control cohort, IL-1B concentrations were significantly increased in patients with depression (*p*=0.005). The preceding authors believe that IL-1B may be thus used as an indicator for the early diagnosis of patients with depression (121). In another study, researchers injected recombinant IL-1B into the murine hippocampus, and this resulted in elevated plasma glucocorticoid levels, which is more likely to induce depression in the presence of chronic stress (122). Of note, IL-1B receptors are widely expressed in the hippocampus (123). According to Mussano et al., the reason for this is that IL-1B is neurotoxic, and promotes neuronal death (124). Studies have also shown that IL-1B levels are significantly increased in PLWH, compared to levels in the general population (125). IL-1B also induces viral replication in cells infected by HIV (126), and the concentration of IL-1B is elevated in the serum of PLWH (127). These findings may predispose PLWH to an increased susceptibility to the development of depression.

#### 4.2.2 Interleukin 6

In addition to IL-1B, IL-6 is of particular importance in the study of depression. Evidence from animal and human clinical studies suggest that elevated levels of IL-6 may interfere with the normal physiological response to stress, and cause depression through activation of the hypothalamic-pituitary-adrenal axis (128). Notably, researchers have demonstrated that an increase in IL-6 levels may further promote depression particularly in individuals affected by comorbidities and/or diseases such as multiple sclerosis (129) and cancer (130). Additionally, studies have shown that IL-6 increases both acute and chronic stress (131). In the study by Gothoda et al., in which ten patients underwent liver resection (an example of acute stress), a significant and positive correlation between acute surgical stress and blood IL-6 levels has been observed (132). In one longitudinal community study assessing the relationship between chronic stress (caring for a spouse with dementia) and IL-6 production, it was observed that the average increase in IL-6 levels in caregivers was approximately four times higher than in noncarers (133). Human and animal clinical studies have also shown that stress may increase IL-6 levels. Elevated IL-6 levels may lead to HPA axis dysfunction, altered synaptic neurotransmission, and reduced neurotrophic factors, which may generate depression (134). Whether it is acute stress, chronic stress, or hyperactivity of the HPA axis, PLWH tend to be predisposed to them all. HIV infection has been demonstrated to induce monocytes and macrophages to express and secrete IL-6. Even with HIV virological suppression, plasma IL-6 levels are significantly higher in PLWH receiving treatment than in uninfected individuals (135). The acute and chronic stress, and the possible long-term neuroinflammation faced by PLWH may lead to higher circulating levels of IL-6, making these individuals much more susceptible to the development of HIV-associated depression.

#### 4.2.3 Tumor necrosis factor-alpha

There is sufficient evidence in the literature to demonstrate that TNF-α is involved not only in the pathophysiology of cancer, but also in immunological functioning and the inflammatory process (136, 137). TNF-α is also an important cytokine involved in HIV-associated depression. TNF-α directly affects neuronal function and survival, regulates the production and secretion of neurotransmitters, and influences (either positively or negatively, depending on physiological or pathophysiological conditions) synaptic transmission (138, 139). TNF-α may also increase blood-brain barrier (BBB) permeability, which may be accompanied by depressive behaviors (138). The following perspectives were cited in a systematic review by Ma et al., viz., a) Plasma TNF-α levels are significantly increased in some patients with depression; b) Antidepressant treatment is associated with a decrease in TNF-α levels; c) Drug-induced elevation of TNF-α levels cause disease behaviors which are similar to depression, and blocking TNF-α alleviates depressive symptoms in animal models and human clinical trials (140). In HIV infection, TNF-α activates NFkB which in turn, upon activation, is translocated in the nucleus, where it binds near the HIV transcription initiation site to promote HIV expression and virion production (141). One study by Okay et al., observed that PLWH who did not receive antiretroviral therapy had higher TNF-α levels than those who received treatment, and both these cohorts had higher TNF-α levels than the normal healthy population (142). At the same time, HIV viral load positively correlates with TNF-α levels in a significant manner (142). Thus, during HIV infection, elevated TNF-α levels disrupts the integrity of the BBB and triggers neuroinflammation, which may substantially increase the likelihood of emergence of HIV-associated depression.

#### 4.2.4 Other neuroinflammatory cytokines

In the HIV context, several studies have observed positive associations between depression and neuroinflammatory cytokines such as interferon gamma (IFN-γ), IL-15, 12, and 18 (143–146).

#### 4.2.5 Chemokines

Chemokines are signaling proteins secreted by certain cells, and have the ability to induce directional chemotaxis in adjacent reactive cells. Over recent years, studies have demonstrated that the relationship between depression and chemokines is relatively close, and that some chemokines have a material impact on depression, e.g., the chemokines CXCL8 (147), CCL2 (148), and CCL3 (149). The relationship between HIV and the chemokine CXCL8 has already been suggested in studies of HIV infection, and most related studies have reported that CXCL8 levels are increased in the serum of PLWH (150). In a review by Mamik and Ghorpade, CXCL-8 was observed to correlate with neurological impairment and HIV disease severity (151). Specifically, the preceding authors have indicated that during HIV-associated neuroinflammation, the levels of CXCL8 are elevated in the brain and in cerebrospinal fluid. In this context, CXCL8 contributes to recruitment of neutrophils and monocytes within the brain. Mamik and Ghorpade believe that targeting CXCL8 and reducing levels of CXCL8 within the brain may reduce immune cell recruitment, and thus prevent certain neuro-inflammatory/degenerative diseases, e.g., Alzheimer’s disease, Parkinson’s disease, HIV-associated neurodegenerative disorder, and possibly depression (151). In a study by Lehmann et al., it was observed that Nef-induced CCL2 expression contributes to HIV brain invasion and neuronal dysfunction, which may also greatly increase the risk of depression (152). Furthermore, other than CXCl8 and CCL2, Schaller et al., have indicated that in the context of inflammation, CCL3 participates in the recruitment and activation of granulocytes by binding to receptors CCR1, CCR4, and CCR5 (153). At present, it is known that CCL3 may be associated with a delay of the progression of HIV disease; however, whether CCL3 is indeed related to HIV-associated depression warrants further study (154). Furthermore, other studies (146, 155, 156) have demonstrated a positive association between interferon-gamma-induced protein (IP)-10 and depressive symptoms. A broader presentation of the implications of cytokines in depression in PLWH has been extensively reviewed by Rakshasa-Loots (157).

## 5 Monoamine neurotransmitters

### 5.1 The monoamine hypothesis

The monoamine hypothesis is currently recognized as one of the fundamental etiological hypotheses for the development of depression (25). The theory argues that depression is caused by a decrease or a functional impairment of monoamine neurotransmitter concentrations within the synaptic spaces of the CNS (158). This theory asserts that depression is closely associated with low levels of monoamines such as DA, serotonin, and NE in the brain (158). These neurotransmitters have a wide range of biological activities, and are involved in many physiological responses of the CNS, such as emotional responses, mental activities, and sleep. When their activity or quantity decreases, the probability of developing depression increases substantially (159).

### 5.2 Monoamine neurotransmitters and HIV-associated depression

#### 5.2.1 Dopamine

DA is the most abundant catecholamine neurotransmitter in the brain. As a neurotransmitter, DA regulates various physiological functions of the CNS. A decrease in DA levels will directly affect the patient’s mood, leading to a poor mental state (160). Over time, anxiety and/or depression may thus manifest (160). In 2004, one study suggested that depression may be associated with low levels of extracellular DA (161). A number of studies have since demonstrated that low levels of DA may be associated with depression. In one study in China, Zhang et al., observed that murine models with low DA levels are more prone to depressive and anxious behavior (162). A similar conclusion was reached in a study by Mallo et al., which observed that murine models of depression have lower levels of extracellular DA (163). Many similar studies exist in the literature, all of which point to the close relationship between low levels of DA and depression (160, 164, 165).

DA remains a factor that warrants discussion in the study of the pathogenesis of HIV-associated depression. Recent studies have demonstrated that HIV infection and psychoactive substances have the ability to affect the integrity of the basal ganglia (BG), which suggests that dopaminergic system dysfunction may be a potential mechanism for the development of HIV-associated depression (166). Saloner et al., used the Baker Depression Inventory-II (BDI-II) to assess the severity of current depression by recruiting 225 participants and measuring their DA levels in cerebrospinal fluid (167). These investigators observed that when DA levels in CSF are decreased, the incidence of depression in PLWH increased (167). In another study by Goulding et al., a significant reduction in tyrosine hydroxylase (TH) levels was observed in the brain of PLWH (168). TH is the rate-limiting enzyme for DA synthesis (168). The conclusions of both of the preceding studies suggest that HIV-associated depression may be closely associated with low levels of DA in the brain.

Furthermore, Kumar et al., have observed that HIV has a wide range of influence in dopaminergic areas of the brain, such as the frontocortical area, the basal ganglia, the caudate, the putamen, the globus pallidus, and importantly, in the substantia nigra, the main site of DA synthesis (169). In the early stages of HIV infection, infected monocytes migrate across the BBB, infiltrate the brain, and infect microglia (170), causing microglial dysfunction (171). After induction of the HIV-1 Tat protein, both the microglia and the functioning of the DA system are altered. Specifically, Tat simultaneously reduces the number of microglia and DA neurons in the substantia nigra pars compacta (172). Subsequently, HIV-1 viral proteins disrupt microglial proteins and receptors that regulate microglia-mediated phagocytosis of neurites, and pre- and post-synaptic phagocytosis (173). Finally, HIV-1 viral proteins may alter the bidirectional relationship between the dopaminergic system and synaptic structures (174). Collectively, evidence indicates that HIV infection leads to microglial dysfunction, which may have major consequences by inducing reduced DA levels and synaptic dysfunction, and this has meaningful ramifications for the development and progression of HIV-associated depression.

#### 5.2.2 5-Hydroxytryptamine

5-hydroxytryptamine (also known as serotonin) was initially isolated in serum, and exists widely in mammalian tissues, especially in the cerebral cortex and in synapses (175). 5-HT is not only an inhibitory neurotransmitter but is also a messenger molecule that can produce pleasant emotions, and affects almost every aspect of brain activity, from regulation of emotions, the adjustment of energy levels, the influencing of memory, to shaping one’s outlook on life (176). As the relative therapeutic efficacy of the selective serotonin reuptake inhibitor (SSRI) class of drugs (such as fluoxetine and paroxetine) for the treatment of depression has become widely acknowledged, the relationship between serotonin levels and depression has received increasing scrutiny (177).

Many studies have confirmed that depression is related to low levels of serotonin (178). In a study by Aleksovski et al., the serum level of serotonin in patients with depression who did not take SSRIs was generally low (179). In an investigation by Park et al., the levels of plasma serotonin in treated mice with depression were significantly increased, and depressive symptoms were also improved (180). Many past studies have determined that abnormal levels of 5-HT and its metabolites are present in the CSF of patients with depression (181). The discovery of multiple serotonin receptor subtypes has further indicated that the serotonin system has an association with the pathogenesis of depression. As such, Bhatt’s study has indicated that in the CNS, 5-HT3 receptors (5-HT3R) are located in regions which have significance to the vomiting reflex, the perception of pain, the reward system, cognition, and depression and anxiety control (182). Recently, Zięba et al., have pointed out that 5-HT2A receptor promotion has an antidepressant effect (183).

In PLWH, the levels of 5-HT become significantly reduced (184). As an essential amino acid, tryptophan (TRP) participates in the kynurenine (KYN) pathway (185) through indoleamine-2,3-dioxidase (IDO) (186), in addition to participating in the production of new proteins and serotonin *in vivo* (187). The activity of IDO may be enhanced by inflammatory cytokines (188). Interestingly, when humans are infected by HIV, the induced neuroinflammation increases the activity of IDO (189), and an increased amount of tryptophan is metabolized to KYN (190), resulting in insufficient raw material to produce 5-HT. It is worth mentioning that many PLWH treated with ART continue to suffer from HIV-associated neurocognitive impairment, which may well be associated with an increase in IDO activity and KYN production, and a decrease in 5-HT levels (191). Other mechanisms, such as the depletion of “good” bacteria [butyrate-producing bacteria such as *Akkermansia muciniphila* (192, 193)] in the gut microbiome and the relative depletion of enteroendocrine cells in the gut [which has been reported during HIV infection (194)] may also be responsible for the low levels of serotonin observed in PLWH. Additionally, Shah et al., have demonstrated that at synapses, PLWH may display upregulated levels of serotonin transporters which further diminish levels of serotonin (195), suggesting that the relative lack of serotonin may be a possible mechanism explaining the onset of depression.

#### 5.2.3 Noradrenaline or norepinephrine

NE is a common neurotransmitter. Adrenaline is converted to NE, is synthesized in presynaptic membrane vesicles, and is released into the synaptic junction (196). While this process requires calcium ions, the rate of NE synthesis is also regulated by calcium ions (197).

Researchers believe that depression occurs in an individual when norepinephrine levels are diminished, and the use of drugs which consume catecholamines may induce the development of depression (198). As a clinical example, it is known that the antihypertensive drug, reserpine, may consume norepinephrine in the synaptic cleft, and subsequently may cause depression (199). N-methyl-d-aspartate (NMDA) receptor antagonists may promote the release of NE from synaptic vesicles and reduce the reabsorption of NE, thus alleviating depression (200). It is also known that long-term treatment of patients with depression with the tricyclic antidepressant, imipramine, may cause blockage of the reabsorption of norepinephrine by the presynaptic membrane, thus promoting an increase in norepinephrine concentration within the synaptic cleft, and improving symptoms and signs of depression (201). Also, measurements of levels of 3-methoxy-4-hydroxyphenylglycol (MHPG), which is a metabolite of NE in the brain, may be used as a predictive marker to evaluate the occurrence and development of depression (202).

Among studies related to the pathogenesis of HIV-associated depression, there are a few studies in the literature concerning the association between the noradrenergic system and the pathogenesis of HIV-associated depression. Studies related to norepinephrine or serotonin and their association with HIV-associated depression are rare. Interestingly, one prospective study by Ironson et al., observed that higher levels of peripheral norepinephrine are associated with accelerated HIV disease progression, as manifested by an increased plasma viral load and decreased CD4+ T-cell counts (203). However, the specific mechanism whereby this may occur remains nebulous. Another study by Saloner et al., has indicated that HIV infection is independently associated with excess norepinephrine in the patient’s CNS, and that excess noradrenergic stimulation induces damage to the hippocampus and the prefrontal lobes, making it more likely to develop depression (204).

Increases in NE concentrations may exacerbate neuroinflammation during HIV infection, even among successfully ART-treated PLWH (204); however, further directed research is warranted in order to determine which specific cellular and cytokine components are involved in the consequent neuroinflammation induced by increases in NE concentrations in the brain.

#### 5.2.4 Other neurotransmitter systems

It is now understood that glutamatergic dysfunction may also lead to the progression of depressive symptoms. This theory emerged from studies using animal models in which the authors demonstrated that HIV proteins (Tat and gp120 for example) may directly and indirectly enhance glutamatergic signaling (205, 206). Consequently, the increased glutamate release and reduced uptake may lead to neuronal injury, which may potentially explain the mechanisms whereby glutamate is associated with HAND and depressive symptoms (207). Lastly, the ability of the brain to adapt and change, which is referred to as neuroplasticity, may be a significant factor as well. Indeed, neuroplasticity may play a role in changes seen in neuroinflammatory factors as well as neurotrophic factors, including neurotransmitters, and therefore contribute to depression and cognitive impairment in PLWH (208, 209). HIV is known for its ability to induce neurological problems such as HAND (210). The development of HAND potentially results from the neuroplasticity that is subsequent to changes orchestrated by HIV infection, which ultimately may provoke the development of depressive symptoms.

## 6 Antiretroviral therapy

Up until the present time, there is no specific available drug or combination of drugs that can cure HIV infection or AIDS (211). In clinical practice, modern ART is usually employed to control and suppress HIV infection. In practical terms, this translates to the regular and lifelong consumption of several (usually three or four) different antiretroviral drugs daily. However, due to the complex modern ART drug combinations now utilized, ART administration tends to induce a series of adverse reactions which materially reduces the quality of life of ART-treated patients. Some antiviral drugs may cause serious neuropsychiatric complications, such as irritability, cognitive disorders, sleep disorders, and even depression (212).

Efavirenz, one of the most widely used non-nucleoside reverse transcriptase inhibitors (NNRTIs), is a common component of modern ART regimens, and is usually used in combination with two nucleoside reverse transcriptase inhibitors (NRTIs). However, the growing concern with respect to its adverse reactions (213) have sometimes led to the substitution of Efavirenz by other drugs in the initial treatment selection, or the conversion of the patients ART regimen to a non-Efavirenz regimen in ART-experienced patients (214). The reason for this is that the most common adverse manifestation experienced by patients receiving Efavirenz treatment are neuropsychiatric reactions (215, 216), which range from short-term effects, such as nightmares, dizziness, insomnia, tension, and attention deficit, to long-term serious symptoms, which include depression, suicidal ideation, and even overt psychosis (217). The sleep disorders and clinical depression caused by long-term use of Efavirenz is one of the most common reasons for patients to cease using this drug.

In 2007, Raltegravir was the first drug of the integrase strand transfer inhibitor (INSTI) class of antiretrovirals to be approved for use, with Elvitegravir and Dolutegravir subsequently being approved for use in 2012 and 2013, respectively (218, 219). These drugs inhibit retroviral integrases, which integrate viral DNA transcripts (via reverse transcription synthesis) into the host cell genome. It is speculated that their overall tolerance may be superior, compared to reverse transcriptase inhibitors (220). However, they may have an impact on the nervous system and have the capacity to induce depression, especially with treatment with Dolutegravir (221). In a recent cohort study involving more than 6,400 patients in different countries, about 3.5% (ranging from 1.4-7.2%) of patients receiving Dolutegravir treatment were observed to actively choose drug withdrawal as a direct consequence of the emergence and manifestation of neuropathic adverse events (222). This drug withdrawal rate was also observed to be higher than those of separate randomized clinical trials utilizing other INSTIs, such as Elvitegravir or Raltegravir (222).

The mechanisms underlying the effects of NNRTIs, INSTIs and other antiretroviral drugs on the CNS include: (i) provoke both oxidative stress and mitochondrial toxicity (223, 224); (ii) disrupt synaptic connections and alter neurotransmitter (acetylcholine) release (225); (iii) induce the release of proinflammatory cytokines (226–230); and (iv) inhibit the DNA polymerase gamma activity and cause mitochondrial toxicity and oxidative stress (231, 232). With the recent progressive implementation of long-acting ART, it is believed that the effects of these newer ART regimens on depression will be considerably reduced. Indeed, long-acting ARTs offer tremendous opportunities to (i) improve HIV treatment adherence, (ii) reduce the onset of opportunistic infections, (iii) reduce risks of drug–drug interactions (DDIs), (iv) reduce toxicity, and (v) reduce the stress and stigma related to compliance with the necessarily rigid daily ART treatment (233–236). Collectively, the preceding advantages may potentially help to mitigate the onset of HIV-associated depression subsequent to ART administration. However, only future evidence-based data will assist in appreciating the real effect that long-acting ART has on the depressive symptoms seen in PLWH.

## 7 Additional factors

### 7.1 Substance (or drug) use (or abuse)

Other than HIV mediation, it is worth noting that drug users with HIV, display a higher prevalence of depression (compared to other PLWH) (18, 237). The mechanisms surrounding the onset of depressive symptoms in these patients are likely to be related to the altered stress response resulting from chronic drug use (238). Indeed, recreational drug use and abuse induce alterations to the stress and reward pathways. Of note, acute administration of alcohol, marijuana, cocaine, and amphetamines activates (i) brain reward pathways (referred to as mesocorticolimbic dopaminergic systems), and (ii) brain stress pathways (including the corticotropin release factor (CRF)-HPA axis and autonomic nervous system pathways). Additionally, elevated ACTH and corticosterone levels have been reported in the plasma of recreational drug users (239, 240). Decreased levels of cortisol have been noted in cases of opiate consumption (241). Over time, regular and chronic consumption of recreational drugs induces addictive behavior, which may lead to the development of depressive symptoms and compulsive behaviors associated with drug seeking (242). In terms of mechanisms related to these outcomes, it is necessary to recall that recreational drugs are also stressors which increase the levels of GCs as well as the release of dopamine (243, 244). However, chronic release of GCs may inhibit dopamine synthesis, particularly when the HPA-axis is altered (245). Thus, to achieve a dopamine reward, addicted drug users tend to increase their drug consumption in an attempt to force the release of dopamine, which further deteriorates the HPA axis and increases their risk of development of depressive symptoms.

### 7.2 Lack of sleep or poor sleep quality

Gutierrez et al., and Wu et al., have reported in their systematic reviews that 30 to 90% of PLWH experience poor sleep quality (246, 247). Interestingly, people older than 40 years old are particularly affected (246). In other studies, sleep disturbances (poor sleep quality, insomnia, and shorter total sleep duration) have been reported among PLWH (248, 249). Thanks to recent investigations, sleep disturbances are becoming recognized as a potential cause of mental health disorders seen in PLWH (250, 251). In a recent study, Daubert et al., have further demonstrated that poor quality of sleep is highly prevalent among women living with HIV and HIV negative women presenting depressive and anxiety symptoms (252), suggesting that attention to sleep quality should be one of the priorities of the clinical management of chronic HIV.

The mechanisms whereby the lack of sleep or poor sleep quality may induce the development of depression during HIV infection are not fully understood. However, it is known that in the general population and in PLWH, markers of inflammation or immune activation (such as TNFα, CRP, IL-6) are closely associated to both depression (253) and sleep quality (254). As indicated previously, TRP participates in the KYN pathway (185) through IDO (186). Importantly, TRP is also a precursor of melatonin, which is an important factor in depression (255). Thus, in a context of HIV infection where inflammation increases the activity of IDO (189) resulting in significant diminution of TRP (256), melatonin synthesis may be affected leading to lack of sleep and an increased risk of depression. Evidence collated by Cho et al., indicates that only depressed patients display an association between poor sleep quality and kynurenine metabolism (257). Interestingly, they have also found that poor sleep quality is also associated with increased C-reactive protein levels in this category of participants, which reinforces the role played by inflammation in depression (257). HIV infection is well known for establishing a chronic state of inflammation and triggering the activity of IDO (258). Therefore, it is quite legitimate to consider the complex relationships between inflammation or immune activation, sleep quality, and depressive symptoms as one of the pathways which explains how poor quality of sleep may induce depression in PLWH.

### 7.3 Undernutrition

HIV infection is also associated with undernutrition (with commonest cases encountered in low- and middle-income countries) (259), and evidence suggests that these entities promote each other (260, 261). As indicated by Alebel et al., undernutrition represents one of the major contributors to HIV/AIDS-related deaths, and promotes the emergence of opportunistic infections (262). Investigations conducted in Tanzania (263) and Ethiopia (262) have reported that PLWH present high levels of nutritional insufficiency, evaluated at 27.7% and 26%, respectively. Recently, in their meta-analysis, Seid et al., have found that 23.74% of HIV-positive adults using ART in sub-Saharan Africa are undernourished (264). Undernutrition is associated with (i) anemia, (ii) loss of appetite, poor nutritional absorption, increased energy need, and (iii) loss of weight and a skinny appearance (259). Unfortunately, loss of weight and a skinny appearance may lead to self-stigmatization, social isolation, and depression.

Interestingly, it is also worth noting that some metabolic parameters and nutrients have been proposed as markers of depression. As such, high triglycerides, low high-density lipoprotein cholesterol (HDL-C), and low hematocrit levels have been commonly observed in patients with depression (265). Particular attention on these metabolic parameters in PLWH may be necessary for confirming their roles in depression during HIV infection. That said, a lack of vitamin B consumption (particularly B12, B9, and B6) (266, 267) has been noted in patients with major depressive disorder. A positive association between (i) low vitamin D serum levels and depression and (ii) poor outdoor exposure to sunshine and depression has also been observed by Anglin et al. (268), and Penckofer et al. (269),, respectively. Similarly, low circulating levels of omega 3 polyunsaturated fatty acid has been associated with major depressive disorder (270). In terms of minerals, depression has been noted in people [particularly women (271)] having an insufficient intake of (i) calcium, iron, magnesium, and zinc (272), as well as (ii) potassium, phosphorus, and copper (271). Interestingly, these nutrients are vital for monoamine synthesis, neuroinflammation control, neuroprotection, and for the synthesis of growth factors (273). Thus, undernutrition may be considered as an epigenetic factor influencing depression. In the context of HIV infection, it is therefore legitimate to assume that undernutrition may foster a fertile milieu which encourages the development of depression.

**Figure 1:**
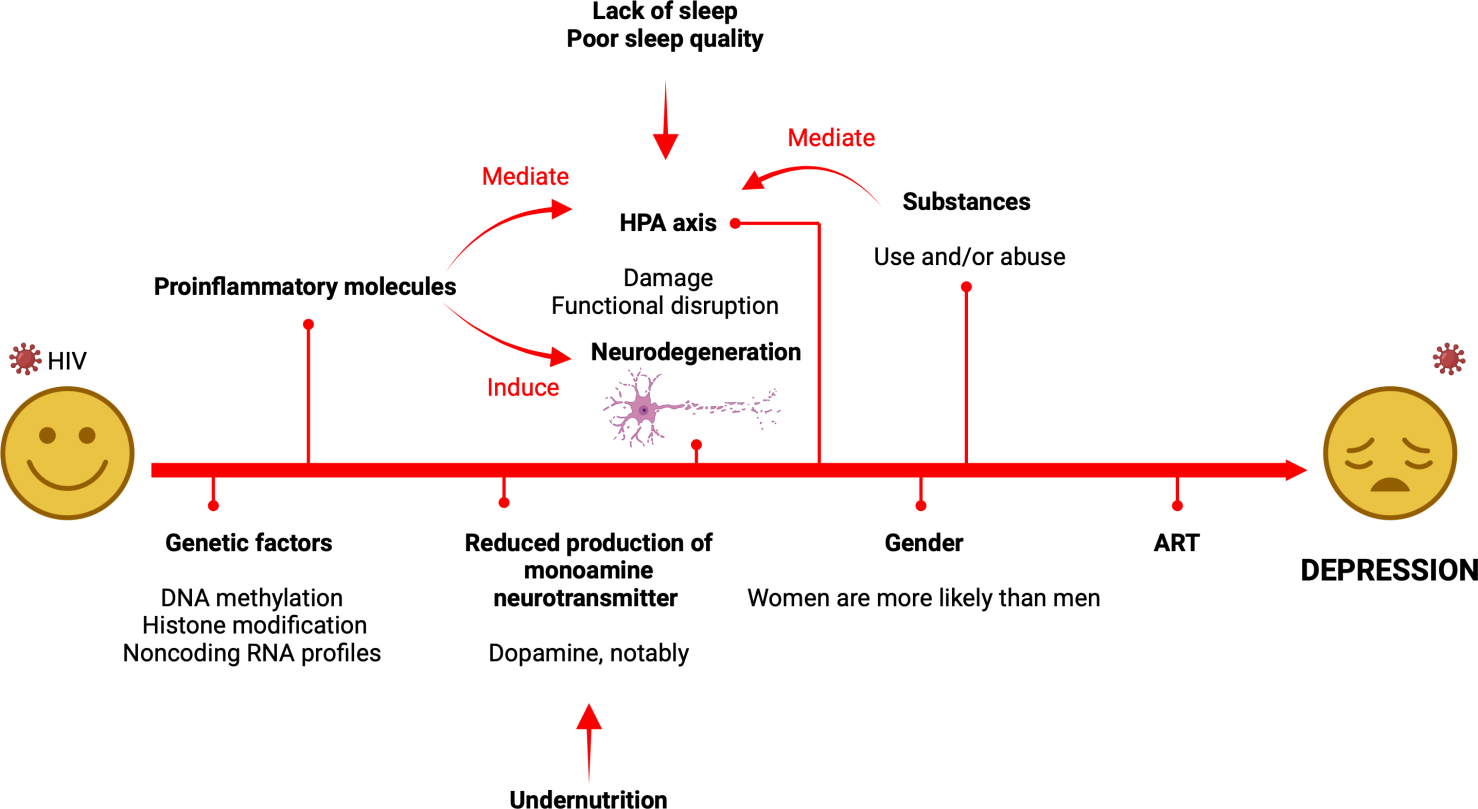
Summary of the factors and mechanisms involved in the pathogenesis of HIV-associated depression. It should be noted that the psychosocial framework of depression, which is not provided in this visual, also plays a significant role as it contributes to the alteration of the HPA axis. The main mechanisms responsible for depression include HPA-axis dysfunction, inflammation/neurodegeneration pathways, and neurotransmitter systems which are significantly perturbed.

An overall representation of the factors involved in the pathogenesis of HIV-associated depression is shown in **Figure 1**. Likewise, **Table 2** summarizes key studies, their methodologies, and findings used to discuss the influence of biological factors in the development of depression in PLWH.

**Table 2:** Biological factors that influence the development of depression in PLWH.

Factors	Ref		Aim or methodology	Findings	Gaps
Genetic	(70)		Compilation of data on neurocognitive-related diseases such as Alzheimer’s disease, amyotrophic lateral sclerosis, age-related macular degeneration, ataxia, dementia, myotonic dystrophy, epilepsy, glaucoma, Huntington’s disease, multiple sclerosis, Parkinson’s disease, and prion disorders.	miR-9-5p, miR-21-5p, the miR-29 family, miR-132-3p, miR-124-3p, miR-146a-5p, miR-155-5p, and miR-223-3p are dysregulated most often in these diseases. These noncoding RNA disorders may promote the development of neurocognitive-related diseases.	More studies of this kind are needed.
	(71)		Review of the literature on the roles of miRs in HIV infection.	miRs play a major role in HIV immunopathogenesis.	The role of miRs in the development of HIV-associated depression was not explored.
	(72)		Utilization of mammalian model (mice) to investigate the mechanism whereby HIV-Tat perturbs neuronal activity.	HIV tat disrupts neuronal activity by blocking the regulation of the level of expression of miRs (including mir-128) in primary cortical neurons.	The investigations were conducted on mice only.
	(73)		Utilization of human neuronal cell line to investigate the role of HIV Vpr in the development of neurocognitive disorders.	Vpr contributes to the development of neurocognitive impairment by dysregulation of the levels of several miRs (for example miR-34a) and their target genes (e.g., CREB, the target gene of miR-34a).	This study did not specifically address the particular case of depression.
	(74)		Explore the role of miRs in the development of HIV-associated dementia.	miR-21, miR-142-3p, and miR142-5p are significantly dysregulated in the caudate nucleus and hippocampus and striatum brain samples of PLWH	The roles of miRs in the development of depression were not addressed.
	(6)		Compared analysis of miRs expression in PLWH with and without HAND.	The levels of miR-500a-5p, miR-34c-3p, miR-93-3p, and miR-381-3p were elevated in the brain of PLWH with and without HAND. These specific miRs may lower the level of peroxisome protein, which may lead to neuronal dysfunction in the CNS.	Further investigations are needed regarding the implication of the miRs in depression.
	(76)		Case-control study comprising 86 HIV associated dementia (HAD) cases and 246 non-HAD cases testing seven candidate gene polymorphisms related to HIV-associated depression.	The SNP in the candidate gene PREP1 was significantly different among the genotypes of all study cases and the control group.	Further investigations are needed regarding the implication of the candidate gene PREP1 in depression.
HPA axis	(94)		Descriptive cross-sectional study on the occurrence of hypocortisolism in 350 PLWH.	108 participants had serum cortisol levels below 100μg/L. After administration of 1μg of ACTH, only 57 participants had serum cortisol levels below 180 μg/L.	The direct relationship between hypocortisolism and depression in PLWH was not addressed
	(95)		Autopsies performed on 88 AIDS patients who succumbed to the disease.	Damage of HPA axis as 10 patients had extensive necrosis and/or focal fibrosis in the anterior pituitary.	HIV induced the damage of HPA axis, but the consequence of this in the onset of depression were not explored.
	(98)		Observational study investigating Cortisol resistance in 9 PLWH.	HIV provoked an adrenal cortical dysfunction. As such, the affinity of the GR and its ligand in peripheral leukocytes decreased significantly, and the number of receptors increased.	To be further investigated as there are few recent such reports. This may indicate that this situation is, in itself, rare, or may be related to an unknown cryptic mechanism.
	(99)		Case-control study on the HIV related damage of the HPA-axis.		
	(100)		A case of a 45-year-old man with HIV-hepatitis C virus co-infection treated with raltegravir and darunavir/cobicistat.	Glucocorticoid levels are significantly elevated in HIV patients with Cushing’s syndrome.	The role of adrenal cortical dysfunction subsequent to HIV infection in the onset of depression is warranted.
Pro inflammatory cytokines	IL-1B	(121)	Compared analysis of subclinical atherosclerosis markers, inflammatory profile, and BDNF production in patients with depression (34), bipolar affective disorder (43), and controls (41).	Compared to patients with bipolar disorder and the control cohort, IL-1B concentrations were significantly increased in patients with depression.	Study population was HIV negative.
		(122)	This study investigated whether IL-1 beta mediates long-term changes in HPA activity and studied the cellular regulation of the anterior pituitary.	Injection of recombinant IL-1B into the murine hippocampus resulted in elevated plasma glucocorticoid levels. This likely induces depression in the presence of chronic stress.	Experiments were conducted on mice.
		(125)	A systematic review of the association between neuroinflammation and HIV neurocognitive impairment and neuropathology from studies investigating post-mortem brain tissue.	IL-1B levels are significantly increased in PLWH, compared to levels in the general population.	This study did not provide direct evidence on how IL-1B induces depression in PLWH.
	IL-6	(129)	This study investigated the possible connection between IL-6, the acute phase of multiple sclerosis, and depression.	Increase levels of IL-6 are observed during the acute phase of the disease, especially when depression is detected.	Study population was HIV negative.
		(274)	Utilization of mammalian model (mice) to investigate the role of IL-6 in stress response.	IL-6 increases both acute and chronic stress.	Experiments were conducted on mice.
		(135)	Compared analysis of biomarkers for inflammation, altered coagulation, and monocyte activation between PLWH and HIV negative controls.	Plasma IL-6 levels are significantly higher in PLWH receiving treatment than in HIV negative individuals.	This study did not provide direct evidence on how IL-6 induces depression in PLWH.
	TNF-α	(140)	Review of the literature on the roles and therapeutic applications of TNF-α in major depressive disorder.	Plasma TNF-α levels are significantly increased in some patients with depression; b) Antidepressant treatment is associated with a decrease in TNF-α levels; c) Drug-induced elevation of TNF-α levels cause disease behaviors which are similar to depression, and blocking TNF-α alleviates depressive symptoms in animal models and human clinical trials.	The particular case of PLWH is not considered.
		(142)	Compared analysis on the cytokine levels between PLWH versus negative controls.	Compared to treated PLWH, those who did not received ART display significantly higher levels of TNF-α. HIV viral load positively correlates with TNF-α levels in a significant manner.	The role of TNF-α in the manifestation of depression was not explored.
	IFN-γ, IL-15, IL-12, and IL-18	(143–146)	Investigations on potential associations between mental health, depression or mood disorders and neuroinflammatory cytokines in PLWH.	Positive associations between depression and neuroinflammatory cytokines.	Despite the positive associations found; the biological mechanisms whereby these proinflammatory cytokines trigger the onset of depression need to be further addressed.
Monoamine neurotransmitters	Serotonin	(179)	Case-control study detecting and exploring the mechanisms of serotonin reuptake inhibitor-induced changes in platelet reactivity in non-responding patients with Recurrent Depressive Disorder and life-long exposure to antidepressants.	The serum level of serotonin in patients with depression who did not take selective serotonin reuptake inhibitors was generally low.	Study population was HIV negative.
		(180)	Observational study evaluating the antidepressant effects of *Gyejibokryeong-hwan* in a mouse model of reserpine-induced depression.	The levels of plasma serotonin in treated mice with depression were significantly increased, and depressive symptoms were also improved.	Experiments were conducted on mice.
		(189)	Indoleamine-2, 3-dioxygenase and other interferon-gamma-mediated pathways in PLWH.	Neuroinflammation increases the activity of IDO in PLWH. According to Murray et al. (190) this results in insufficient raw material to produce serotonin in PLWH.	Further evidence-based studies are needed.
		(195)	Utilization of SIV-infected macaque (as animal model of HIV) to longitudinally image serotonin transporter (SERT) expression before and after inoculation.	At synapses, PLWH may display upregulated levels of serotonin transporters which further diminish levels of serotonin.	Further investigations of this kind with PLWH are needed.
	Noradrenaline or norepinephrine	(200)	A review of the mechanism of antagonism of N-methyl-D-aspartate receptor by ketamine in treatment-resistant depression.	Depression may be alleviated through administration of N-methyl-d-aspartate (NMDA) receptor antagonists. Thus, they may promote the release of NE from synaptic vesicles and reduce the reabsorption of NE.	The context of HIV infection is not specifically presented.
		(203)	A prospective study examining NE, cortisol, depression, hopelessness, coping, and life event stress as predictors of HIV progression in a diverse sample.	Higher levels of peripheral norepinephrine are associated with accelerated HIV disease progression, as well as depression.	The specific mechanism whereby norepinephrine may trigger depression remains nebulous.
		(204)	Study evaluating associations between cerebrospinal fluid NE and HIV, METH dependence, and neurocognition.	HIV infection is independently associated with excess norepinephrine in the CNS of PLWH, which induces damage to the hippocampus and the prefrontal lobes.	The mechanisms and signaling pathways leading to depression in PLWH should be further explored.
Antiretroviral therapy	(215–217)		Literature review on effects of efavirenz on the CNS of PLWH.	PLWH receiving efavirenz experience neuropsychiatric reactions	The root-cause of the mechanisms whereby efavirenz causes sleep disorder and clinical depression need to be further explored.
	(222)		Literature review on the neuropsychiatric adverse events observed with dolutegravir and other integrase strand transfer inhibitors	Emergence and manifestation of neuropathic adverse events subsequent to Dolutegravir administration	Further studies of this kind are needed.
Substance (or Drug) use (or abuse)	(238)		Literature review on the influence of stress and substance abuse on depressive symptoms.	The mechanisms surrounding the onset of depressive symptoms in these patients are likely to be related to the altered stress response resulting from chronic drug use	Further evidence-based studies are needed.
	(18)		A review of the literature on the intriguing associations between HIV/AIS and mental health	Compared to other PLWH, drug users with HIV, display a higher prevalence of depression	Further evidence-based studies are needed.
	(237)		Study on the prevalence and predictors of depressive symptoms in men living with HIV who inject drugs	Compared to other PLWH, drug users with HIV, display a higher prevalence of depression	The mechanisms behind this phenomenon need more attention.
Lack of sleep or poor sleep quality	(252)		This is an observational study exploring the association of poor sleep with depressive and anxiety symptoms in women with and without HIV.	Poor quality of sleep is highly prevalent among women infected by HIV and HIV negative women presenting depressive and anxiety symptoms.	The study only focused on women with and without HIV.
	(253, 254)		Studies exploring the relationships between inflammatory markers, depression, and sleep quality.	Markers of inflammation or immune activation (including TNFα, CRP, IL-6) are closely associated with depression and sleep quality.	The mechanisms whereby poor sleep quality induces depression in PLWH need to be further investigated.
Undernutrition	(262, 263)		Meta-analysis on nutritional conditions of PLWH.	PLWH present high levels of nutritional insufficiency, evaluated at 27.7% (in the study by Sunguya et al) and 26% (in the study by Alebel et al).	The consequences on the development of depression were not evaluated.
	(265)		This study assessed whether there are significant associations of depressive disorder with biochemical and obesity indices.	High triglycerides, low high-density lipoprotein cholesterol, and low hematocrit levels have been commonly observed in patients with depression	The role of these metabolic parameters in depression during HIV infection should be further explored.
	(266, 267)		These studies determined whether nutritional and biochemical parameters are associated with depression	A lack of vitamin B consumption (particularly B12, B9, and B6) has been noted in patients with major depressive disorder	Study population was HIV negative.
Psychosocial	(101)		An experience sampling study on 109 PLWH about their experiences of recent acts of discrimination, internalized HIV stigma, avoidance coping with HIV, and recent social support.	PLWH experience social isolation, discrimination, stigmatization, and other negative psychological emotions.	How these circumstances modulate HPA axis and how they may therefore lead to depression should be further investigated.
	(102)		Longitudinal study exploring the predictors of anxiety and depression among newly diagnosed people living with HIV.	Proportion of people belonging to socially vulnerable groups is high, and these individuals have an inherently greater risk of developing depression even before HIV infection.	The mechanisms whereby internalized stigma and its effects act in onset of depression were unexplored.
	(275)		Machine learning combined with inferential methods to identify novel mental health phenotypes among PLWH and the underlying explanatory features	PLWH with mental health disorders, including anxiety and depression have post-traumatic stress disorder subsequent to sexual abuse in their childhood.	Early life experiences play a significant role in mental health disorders; however, the biological factors and their mechanisms were not addressed.
	(276)		Study of HIV-Related Stress Experienced by Newly Diagnosed PLWH	HIV-related stress experienced by newly diagnosed patients can lead to depression	The roles of biological factors and their mechanisms were not addressed.

Ref, references.

### 7.4 Psychosocial factors

Lastly, it is worth nothing that in addition to the biological factors and mechanisms extensively expounded in this article, pre-existing psychosocial factors also play a major role in the development of depression in PLWH. In other words, the psychosocial milieu within which the individual exists significantly contributes to the development of mental health dysfunction, or more specifically depression, in PLWH. To illustrate this point, a recent publication by Rubin et al. (275), clearly presents some evidence. Indeed, they demonstrate that with respect to the psychosocial traits of PLWH, 4 clusters of mental health phenotypes in PLWH may be identified. Thus, clusters 1, 2, 3, and 4 designate patients with significant post-traumatic stress disorder (PTSD) symptoms, anxiety, mixed anxiety/depression, and no clinical mental health symptoms, respectively. Interestingly, cluster 1 (significant PTSD) individuals were observed to have an inherently higher rate of childhood sexual abuse compared to (i) cluster 4 (PLWH with no clinical mental health symptoms) and (ii) cluster 2 and 3 (PLWH with anxiety and/or depression who display minimal PTSD symptoms) individuals. Further comparative analysis revealed that compared to cluster 4, those with minimal PTSD symptoms were also observed to have had experienced higher rates of childhood sexual abuse. Although the cases of sexual abuse cannot be generalized to the entire population of PLWH suffering from depression, this study highlights the critical role that early life adversity plays in the onset of mental health disorders in PWLH in adulthood. Similarly, Huang et al. (276), have observed that HIV-related stress experienced by newly diagnosed patients may lead to depression. They have suggested that in order to mitigate the onset of anxiety and depression in these patients, interventions aimed at reducing stress among PLWH should be taken into consideration for the management of known stressors such as discrimination/stigma, fear of infecting others, and excessive attention to physical functions, amongst others. Furthermore, studies have also shown that PLWH (i) experience stressful social circumstances (social isolation, discrimination, stigmatization, and other negative psychological factors) (101) and (ii) tend to belong to socially vulnerable groups (sex workers, economically vulnerable, ethnic minorities, and other vulnerable groups) (102). Collectively, these ‘negative’ circumstances may modulate and facilitate the onset of depressive symptoms via a series of stress responses which potentially alters the homeostatic functioning of the HPA axis.

## 8 Conclusions and perspectives

Due to its high prevalence and severity, clinical depression in PLWH has evolved into a global public health problem. HIV-associated depression most likely results from enigmatically complex interactions amongst genetic, biological, and psychosocial factors. HIV infection is often accompanied by HPA axis damage and functional disruption, a large release of inflammatory cytokines, a reduction of monoamine neurotransmitters (especially DA), and increased neuronal death, all of which may increase the incident rate of HIV-associated depression. Additionally, some commonly used modern antiretroviral drugs (especially Efavirenz and Dolutegravir) may also have a negative impact on the CNS of PLWH, making patients more susceptible to the development of depression. The causes of HIV-related depression are, thus, complex and multifactorial, among which the release of cytokines, overactivation of the HPA axis, and changes in levels of monoamine neurotransmitters play key roles. Over the long term, treatment of depression in HIV/AIDS patients and the psychological monitoring of these patients should thus be optimized. With respect to ART treatment, it would be interesting to investigate the effects of long-acting antiretroviral therapy (which is a novel, efficacious, and much more robust therapeutic approach against HIV infection) on depression. In other words, does a switch to long-acting therapy favor or mitigate the onset of depression in PLWH? Only future studies will provide clearer answers to this question. Moreover, clinicians should be aware of the potential emergence of depression in PLWH, and at the same time anticipate and actively draft individualized patient treatment plans to improve the quality of life of all patients afflicted by HIV-related disease. Interventions should thus be directed towards those who are more likely to be affected by depression. As such, it is critical to indicate that there are differences between women and men with respect to the incidence of depression. Indeed, it seems that women infected by HIV are more likely to develop depression than men infected by HIV (277). Among the reasons for this, structural barriers, internalized stigma, and masculine coping styles are high on the list. Compared to men with HIV, women with HIV experiencing structural barriers seem to be more likely to develop depression through mental health impairment, as reported by Waldron et al. (278),. Besides, Celeste-Villalvir et al. (279), and Waldron et al. (278), have observed that women with HIV, compared to men with HIV, tend to experience internalized stigma, which makes it difficult for them to seek help and care. This context may also lead to depression. Lastly, according to Shi et al. (280),, men with HIV (as a consequence of their masculine coping style) are less likely to seek help when they have depression. Instead, men may express their depression through other symptoms, such as aggression and substance abuse (280). This results in fewer men being diagnosed with depression and consequently, this translates to a higher proportion of women being diagnosed with depression than men.

This narrative review (when compared to other recent related publications) aims to address and elucidate the fundamental mechanisms involved in the emergence of depression in the context of HIV infection. As such, factors favoring the emergence of depression are reported on, and the mechanisms potentially involved in the pathogenesis of depression in PLWH are presented as well. However, as a narrative review, this article does not provide original evidence-based data. Thus, the observations and the conclusive remarks remain subjective, and only further observational or evidence-based studies will provide stronger and more credible observations and conclusions to the topic at hand. Moreover, the selection of reference articles used in this study may be construed to be prejudicial, as article selection is inherently based on the subjective opinions and preconceptions of the specific authors involved in the study. Indeed, due to the very large number of publications concerning this topic present in the contemporary literature since the beginning of HIV epidemic, some may consider that the present review of the literature may not be robust enough, may be inappropriate, or is incomplete.

Notwithstanding these limitations, the present article provides a deeper appreciation of the roles of the HPA axis, inflammation, and monoamine neurotransmitters in the manifestation of depression in PLWH. Although there are several factors associated with depression in PLWH, most of these factors are closely related to HPA axis functionality, inflammation, and neurotransmitter release. As such, orienting future interventions which target restoration of the HPA axis, reduction (or mitigation) of the inflammatory syndrome, and regulation of the expression of dopamine (for instance) may be reasonable and viable approaches to treat depression in PLWH. The regulation of inflammation and/or the expression of neurotransmitters such as dopamine may be achieved pharmacologically. Regarding the restoration of the HPA, two strategies (used separately or in tandem) exist (281). Firstly, the restoration of the HPA axis may be achieved through lifestyle adjustments. For example, these adjustments can be oriented toward addressing (i) the underlying psychological problems, (ii) the nutritional habits, (iii) the quality of sleep, and associating (iv) regular physical activity to ultimately mitigate the effects of chronic stress on the HPA axis. Secondly, other than lifestyle adjustments, a pharmacological approach may be explored. This mainly relies on administration of corticosteroids followed by an assessment of HPA function. However, further work is necessary in the area of the epigenetics of depression in the context of HIV infection. It would be intellectually interesting, for instance, to study the levels of methylation of the genes that are involved in depression, and also the expression of miRs and their implications in the manifestation of depression in PLWH.

Contemporary literature indicates that considerable progress has been made in biomedical research concerning the pathogenesis of HIV-related depression. Nonetheless, it is necessary to redouble our efforts to fundamentally and accurately describe this specific subtype of clinical depression, and to clearly elucidate its etiology, pathophysiology, evaluation, treatment, and prognosis in a more robust and meaningful manner. Further directed research is therefore warranted in order to achieve these lofty but achievable objectives.
